# Das komplizierte kindliche Mittelohrhämangiom

**DOI:** 10.1007/s00106-022-01261-y

**Published:** 2023-01-12

**Authors:** Miray-Su Yılmaz Topçuoğlu, Cornelia Hornberger, Carlota Lucena-Porcel, Ingo Baumann

**Affiliations:** 1 Hals-, Nasen- und Ohrenklinik, Universitätsklinikum Heidelberg, Im Neuenheimer Feld 400, 69120 Heidelberg, Deutschland; 2 Pathologisches Institut, Universitätsklinikum Heidelberg, Im Neuenheimer Feld 224, 69120 Heidelberg, Deutschland

**Keywords:** Gefäßtumor, Hördiagnostik, Kind, Schallleitungsschwerhörigkeit, Paukenerguss, Vascular tumor, Auditory testing, Child, Conductive hearing loss, Otitis media with effusion

## Abstract

Mittelohrhämangiome bei Kleinkindern sind selten. Die Kasuistik beschreibt einen dreijährigen Jungen mit einseitiger Hörminderung und rezidivierenden Paukenergüssen durch ein Mittelohrhämangiom. Dieses verursachte eine Sinusvenenthrombose, Osteomyelitis und Mastoiditis. Eine interdisziplinäre Behandlung erfolgte. Auch gutartige Läsionen des Mittelohrs können schwerwiegende, lebensbedrohliche Komplikationen aufgrund der engen nachbarschaftlichen anatomischen Beziehungen des Mittelohrs hervorrufen. Jede asymmetrische Hörminderung sollte deshalb stets genauer untersucht werden.

## Abstract (en)

Hemangiomas in the pediatric middle ear are a rare occurrence. We present the case of a 3-year-old boy with unilateral hearing impairment and recurrent otitis media with effusion due to a hemangioma in the middle ear. This caused a venous sinus thrombosis, osteomyelitis, and mastoiditis. Interdisciplinary treatment was performed. This case shows that even benign lesions in the middle ear can cause severe, life-threatening complications due to the close proximity of important anatomic structures in the middle ear. Every case of asymmetric hearing loss should thus be examined in detail.

## Anamnese

Ein dreijähriger Junge wurde in der Pädaudiologie vorstellig mit rezidivierenden Mittelohrentzündungen, chronischem Paukenerguss und negativem Neugeborenen-Hörscreening des linken Ohrs. Das rechte Ohr war beschwerdefrei.

## Untersuchungsbefund

In der Ohrmikroskopie zeigte sich ein Paukenerguss mit mattem Trommelfell auf der linken Seite und ein normaler Spiegelbefund rechts.

## Diagnostik

**Figure 1:**
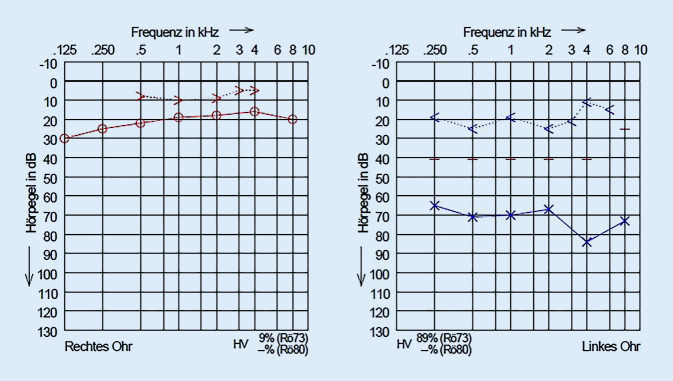

Die Diagnostik ergab im Tonaudiogramm eine deutliche Schallleitungsschwerhörigkeit auf der linken Seite (Abb. 1), ein abgeflachtes Tympanogramm links sowie fehlende otoakustische Emissionen links. Es lag eine weitgehende Normakusis rechts vor.

## Therapie und Verlauf

Bei nicht erfolgreicher sekretolytischer Therapie durch die niedergelassene HNO-Praxis wurde die Indikation zur intraoperativen Ohrmikroskopie mit Parazentese und Paukendrainage gestellt. Intraoperativ zeigte sich das linke Trommelfell rötlich und vorwölbend bei weitgehendem Normalbefund rechts. Nach der Parazentese links kam es zu einer Blutung aus dem Mittelohr, welche mit topischer Applikation von in Epinephrin getränkten Wattetupfern und nach Zuwarten wieder sistierte. Hinter dem Parazenteseschnitt zeigte sich eine weiche rötliche Bindegewebsvermehrung im linken Mittelohr.

**Figure 2:**
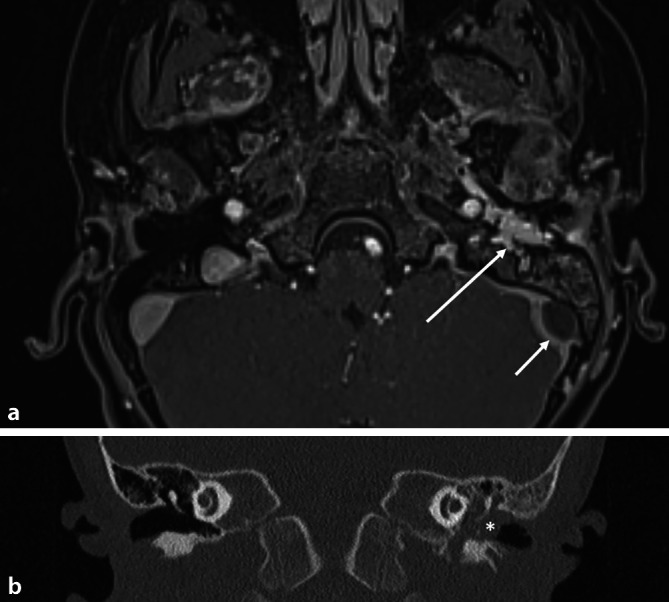

Eine MRT- und CT-Bildgebung wurden im nächsten Schritt durchgeführt (Abb. 2a,b). Hier zeigte sich eine kontrastmittelaffine Weichgewebsvermehrung des linken Mittelohrs. Diese dehnte sich bis zur Tuba auditiva, dem Canalis nervi facialis sowie der kranialen Mastikatorloge und der Schädelbasis inklusive Erreichen der Gefäß-Nerven-Scheide nach anterokaudal aus. Eine Ausdünnung des Tegmen tympani hin zur mittleren Schädelgrube und der Pars tympanica des Os temporale zeigte sich. Zusätzlich wurde anhand der Bildgebung eine asymptomatische Sinusvenenthrombose links sowie eine Osteomyelitis, ausgehend vom linken Mastoid, diagnostiziert. Eine antithrombotische Therapie mit Enoxaparin-Natrium subkutan zunächst therapeutisch zweimal täglich 1 mg/kg Körpergewicht (kgKG), im weiteren Verlauf prophylaktisch mit 20 mg für insgesamt 6 Monate und eine antibiotische intravenöse Therapie mit Clindamycin und Ampicillin/Sulbactam gewichtsadaptiert für zwei Wochen wurden eingeleitet. Hierunter konnte der Sinus sigmoideus erfreulicherweise erfolgreich rekanalisiert werden. Die Osteomyelitis zeigte sich ebenfalls rückläufig, jedoch im protrahierten Verlauf. Deshalb erhielt der Patient durch die pädiatrischen Kollegen zudem als Antiphlogistikum eine orale Naproxentherapie mit einer Dosis von 10 mg/kgKG/Tag für insgesamt 6 Monate.

**Figure 3:**
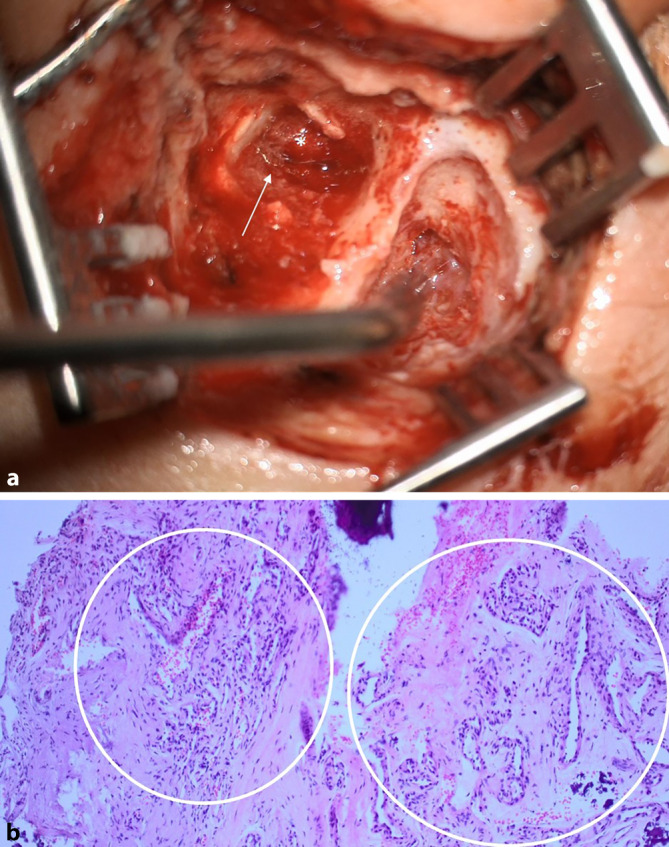

Im nächsten Schritt nach Rekanalisation des Sinus sigmoideus und Befundverbesserung der Osteomyelitis erfolgte 10 Wochen nach der eigentlichen Parazentese eine Tympanoskopie und Mastoidektomie zur Exploration und histologischen Sicherung des Befundes. Intraoperativ zeigte sich eine vaskuläre Läsion, welche sich im gesamten Mittelohr bis zu den benachbarten Strukturen ausdehnte (Abb. 3a). Die histologische Untersuchung ergab in der Zusammenschau der klinischen und radiologischen Befunde die Verdachtsdiagnose eines Hämangioms (Abb. 3b).

Eine Befundresektion wurde mit den Eltern in einer Risiko-Nutzen-Abwägung diskutiert. Von Seiten der Eltern wurde diese abgelehnt bei – abgesehen von der Hörminderung – fehlenden Anzeichen von weiteren Symptomen und behobener Sinusvenenthrombose.

Im weiteren Verlauf, fünf Monate nach der Tympanoskopie und histologischen Sicherung des Befundes, entwickelte das Kind eine ausgeprägte akute eitrige Mastoiditis, sodass eine Mastoidektomie mit Radikalhöhlenanlage durchgeführt wurde. Eine komplette Befundexzision des Hämangioms erfolgte bei akut eitrigem Zustand des Mittelohrs mit Durabeteiligung nicht. Zu diesem Zeitpunkt berichteten die Eltern, dass 4 Monate zuvor eine leichte, fast nicht erkennbare periphere Fazialisparese auf der linken Seite bestanden hatte. Die Fazialisparese war in engmaschiger Beobachtung mit den niedergelassenen Kinderärzten innerhalb von 7 Tagen wieder vollständig rückläufig und somit zum Zeitpunkt der Mastoidektomie nicht mehr vorhanden.

Nach der Mastoidektomie entwickelte der Patient keine weiteren Symptome mehr bis auf die bekannte und bleibende Schallleitungsschwerhörigkeit auf dem betroffenen Ohr.

Bei weiterhin nicht bestehendem Wunsch nach Befundresektion durch die Eltern nach ausführlicher Risiko-Nutzen-Abwägung wurde eine regelmäßige Verlaufskontrolle mittels klinischer Untersuchung sowie bildgebend mit einer MRT des Schädels zunächst alle 6 Monate eingeleitet. In dieser Nachsorge befindet sich der Patient aktuell. Es zeigt sich ein stabiler Befund ohne Dynamik. Bei bleibender Schallleitungsschwerhörigkeit wurde zur ausreichenden Gehörrehabilitation eine Versorgung des Kindes mit einem Knochenleitungssystem in die Wege geleitet.

## Diskussion

Es sind bisher nur wenige Fälle von Mittelohrhämangiomen bei Kleinkindern bekannt [2–5]. Deshalb ist es wichtig, möglichst viele Fallbeispiele zu sammeln und deren Verläufe zu evaluieren.

Mittelohrhämangiome, insbesondere bei Größenprogredienz, können rezidivierende Ohrinfektionen mit einhergehender Hörminderung, aber auch schwerwiegende Symptome wie eine Fazialisparese, Mastoiditis, Sinusvenenthrombose sowie Osteomyelitis hervorrufen [2–5]. Dies zeigte sich auch beim Patienten dieser Kasuistik. Es wurde jedoch auch schon von sich spontan zurückbildenden Mittelohrhämangiomen berichtet [2]. Mittelohrhämangiome stellen zudem eine wichtige Differenzialdiagnose dar bei Tumoren des Mittelohrs [1–5]. Mögliche Differenzialdiagnosen sind beispielsweise Glomustumoren, arteriovenöse Malformationen, aberrante Verläufe der Arteria carotis interna, ein Bulbushochstand, Cholesteringranulome, Meningeome oder auch eine polypoid-entzündlich veränderte Mittelohrschleimhaut [1–5]. Eine gültige Leitlinie oder eine eindeutige Therapieempfehlung gibt es aufgrund der geringen Fallzahl bisher nicht. Mögliche Therapiemodalitäten sind die chirurgische Resektion, die ebenfalls Risiken einer Verletzung wichtiger anatomischer Strukturen birgt, aber auch das konservative Vorgehen mit engmaschigen klinischen und bildgebenden Kontrollen, sofern der Befund stabil ist [2–5]. Das Ziel sollte stets sein, die Komplikationsquote so gering wie möglich zu halten. Eine ausführliche individuelle Risiko-Nutzen-Abwägung gemeinsam mit den Betroffenen sollte dann erfolgen, um zu entscheiden, welche Therapiestrategie gewählt werden soll.

In unserem Fall zeigten sich in dem Jahr nach der notfallmäßigen Mastoidektomie mit Radikalhöhlenanlage eine Verbesserung der Symptome, da der Patient – abgesehen von der Hörminderung – keine weiteren Begleitsymptome und insbesondere auch keine rezidivierenden Ohrinfektionen mehr erlitt. Die Schallleitungsschwerhörigkeit zeigte sich stabil, und die Versorgung mit einem Knochenleitungssystem wurde in die Wege geleitet. Grund für die Befundstabilität könnte sein, dass durch die Mastoidektomie mit Radikalhöhlenanlage eine ausreichende Erweiterung der Mittelohrräume stattgefunden hat, sodass das Mittelohrhämangiom die benachbarten anatomischen Strukturen nicht mehr verdrängte und somit weniger Symptome verursachte.

Da es nur wenige Fallberichte von Kindern mit Mittelohrhämangiomen gibt, sind weitere multizentrisch basierte Untersuchungen notwendig, um optimale Therapiestrategien zu entwickeln.

## Fazit für die Praxis

- Zur Therapieoptimierung bei einem kindlichen Mittelohrhämangiom ist ein interdisziplinäres Zusammenspiel von klinischen, radiologischen und histologischen Untersuchungen essenziell.
- Mittelohrhämangiome können, auch wenn die Entität an sich gutartig ist, schwerwiegende und lebensbedrohliche Komplikationen hervorrufen.
- Es ist daher stets wichtig, eine asymmetrische kindliche Hörbeeinträchtigung detailliert zu untersuchen, da auch seltene Differenzialdiagnosen im Alltag des HNO-Arztes auftreten.
